# Harnessing CRISPR-Cas to Combat COVID-19: From Diagnostics to Therapeutics

**DOI:** 10.3390/life11111210

**Published:** 2021-11-09

**Authors:** Kok Gan Chan, Geik Yong Ang, Choo Yee Yu, Chan Yean Yean

**Affiliations:** 1 International Genome Centre, Jiangsu University, Zhenjiang 212013, China; kokgan@um.edu.my; 2 Institute of Marine Sciences, Shantou University, Shantou 515063, China; 3 Division of Genetics and Molecular Biology, Institute of Biological Sciences, Faculty of Science, University of Malaya, Kuala Lumpur 50603, Malaysia; 4 Faculty of Sports Science and Recreation, Universiti Teknologi MARA, Shah Alam 40450, Malaysia; 5 Laboratory of Vaccine and Biomolecules, Institute of Bioscience, Universiti Putra Malaysia, Serdang 43400, Malaysia; 6 Department of Medical Microbiology and Parasitology, School of Medical Sciences, Universiti Sains Malaysia, Kota Bharu 16150, Malaysia

**Keywords:** coronavirus, COVID-19, isothermal amplification, antiviral, CRISPR-Dx

## Abstract

The coronavirus disease 2019 (COVID-19), caused by severe acute respiratory syndrome coronavirus 2 (SARS-CoV-2), remains a global threat with an ever-increasing death toll even after a year on. Hence, the rapid identification of infected individuals with diagnostic tests continues to be crucial in the on-going effort to combat the spread of COVID-19. Viral nucleic acid detection via real-time reverse transcription polymerase chain reaction (rRT-PCR) or sequencing is regarded as the gold standard for COVID-19 diagnosis, but these technically intricate molecular tests are limited to centralized laboratories due to the highly specialized instrument and skilled personnel requirements. Based on the current development in the field of diagnostics, the programmable clustered regularly interspaced short palindromic repeats (CRISPR)/CRISPR-associated proteins (Cas) system appears to be a promising technology that can be further explored to create rapid, cost-effective, sensitive, and specific diagnostic tools for both laboratory and point-of-care (POC) testing. Other than diagnostics, the potential application of the CRISPR–Cas system as an antiviral agent has also been gaining attention. In this review, we highlight the recent advances in CRISPR–Cas-based nucleic acid detection strategies and the application of CRISPR–Cas as a potential antiviral agent in the context of COVID-19.

## 1. Introduction

Since the coronavirus disease 2019 (COVID-19) was declared a pandemic, the disease caused by novel severe respiratory syndrome coronavirus 2 (SARS-CoV-2) has infected more than 231 million people and claimed 4 million lives worldwide as of 27 September 2021 [1]. The healthcare system in various countries has been stretched beyond its capacity, with some even collapsing under the strain of the pandemic and the implementation of rigorous mitigation efforts to slow down the virus transmission, such as lockdown, travel restriction, and social distancing, have brought catastrophic effects to the global economy and society [2]. Although the majority of COVID-19 cases are mild, the disease can progress rapidly from mild to severe with serious complications including acute respiratory distress syndrome, acute cardiac injury, acute kidney injury, and septic shock [3,4]. Various existing medicines have been repurposed for the treatment of COVID-19 patients, including several protease inhibitors against human immunodeficiency virus (such as lopinavir and ritonavir), but they were minimally efficacious and caused adverse effects in some patients [5]. At present, there is not a single specific antiviral therapy for COVID-19 and symptomatic supportive care remains the mainstay of treatment [6]. Given that asymptomatic and presymptomatic cases have been found to harbor similar viral loads as those who are symptomatic [7,8], the importance of case detection, isolation, and contact tracing to limit the transmission of SARS-CoV-2 cannot be understated.

During the early phase of the COVID-19 pandemic, resources were directed towards high-priority areas that include timely identification of SARS-CoV-2-positive individuals. The ~30 kb genome of SARS-CoV-2 was unraveled in record time and similar to other coronaviruses (CoVs), the positive-sense, single-stranded RNA (ssRNA) genome was found to encode for non-structural proteins, structural proteins (spike (S), envelope (E), membrane (M) and nucleocapsid (N)), and accessory proteins [9]. SARS-CoV-2 enters the epithelial cells of the human host by interacting with the angiotensin-converting enzyme 2 receptor through its S protein that can be functionally divided into two subunits: the receptor-binding S1 subunit and the membrane-fusion S2 subunit [10,11,12]. Availability of the SARS-CoV-2 genome during the early COVID-19 outbreak was instrumental to the successful development of various nucleic acid-based COVID-19 diagnostic, tests particularly real-time reverse transcription-polymerase chain reaction (rRT-PCR), which is deemed as the gold standard molecular method [13]. However, rRT-PCR tends to be restricted to large laboratories and/or reference centers due to the technical intricacy associated with the molecular test. In addition to the skilled personnel and specialized instrument requirements, rRT-PCR typically takes 4–6 h to complete and the turnaround time can be longer than 24 h if sample collection and shipment to a centralized laboratory, batch testing, and laboratory report generation are taken into consideration [14,15].

Compared to rRT-PCR, isothermal amplification methods, which include loop-mediated isothermal amplification (LAMP), recombinase polymerase amplification (RPA), and recombinase-aided amplification (RAA), remove the need for a thermocycler as the amplification process is carried out at a constant temperature, but these methods tend to suffer from non-specific amplification [16]. On the other hand, next-generation sequencing technology offers single-nucleotide resolution but involves the use of a costly sequencer, tedious library preparation, and a post-sequencing bioinformatic pipeline for the analysis of sequencing data [13,17,18]. Lateral flow immunoassays (LFIAs) that detect SARS-CoV-2 antigen or anti-SARS-CoV-2 antibodies are currently being used to complement molecular diagnostic capabilities because the advantages associated with LFIA (i.e., simplicity, portability, speed, and electricity-free operation) make the technology well-suited for point-of-care (POC) settings. Serological-based LFIAs that detect the presence of IgM and IgG against SARS-CoV-2 may provide indication of an active or past infection but are of limited value in diagnosing early infection due to the delay in seroconversion [19,20]. Antigen testing with LFIA is used instead for early case detection as it circumvents the time needed for the body to mount an immune response, but conventional LFIA generally suffers from poor sensitivity and operator bias may occur when the results are visually interpreted.

In recent years, the clustered regularly interspaced short palindromic repeats (CRISPR)/CRISPR-associated proteins (Cas) system has not only advanced the field of genome editing but has also emerged as a promising diagnostic tool and antiviral agent. RNA-guided CRISPR-Cas technology for nucleic acid detection has been hailed as the next-generation POC diagnostics due to the versatility, rapidity, portability, and more importantly, high sensitivity and specificity of the CRISPR-Cas systems [21]. The emergence of pandemic SARS-CoV-2 poses a huge challenge, as little was known about the new pathogen during the initial outbreak, and the subsequent need for novel diagnostic tests to be developed and validated before they could be implemented in different testing sites impeded the rapid containment of the disease. In line with efforts to increase testing accessibility and capacity, the applications of the CRISPR-Cas system in diagnostics as well as prophylactics and therapeutics for COVID-19 are appealing and highly desirable to contain and prevent the further spread of the disease. In this review, we present the latest advances in the CRISPR-Cas-based nucleic acid detection platform for COVID-19, including strategies that were used to simplify the molecular workflow and to enhance the sensitivity and specificity of the CRISPR-Cas system. We also summarize the characteristics of the selected CRISPR-Cas system and highlight the challenges and future directions with regard to POC, prophylactic, and therapeutic applications.

## 2. Molecular Mechanism of CRISPR-Cas

**Figure 1:**
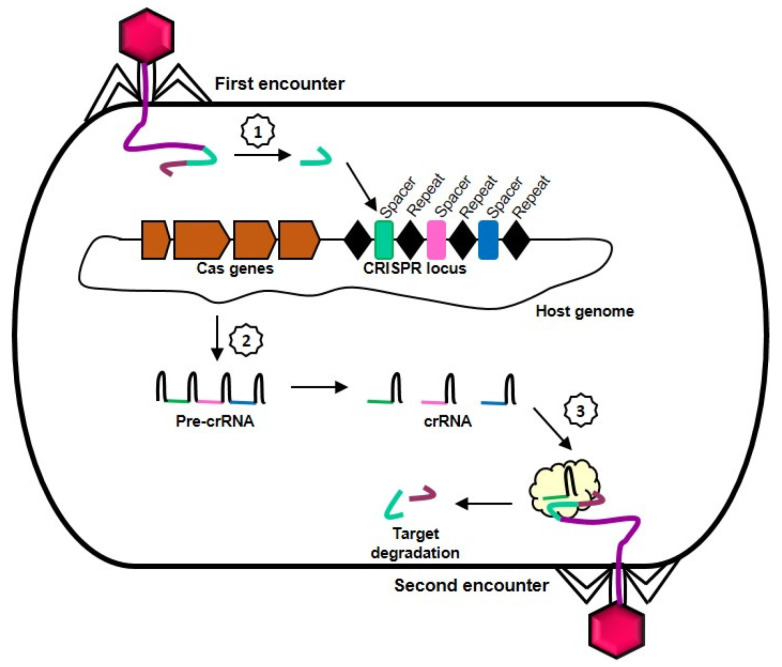
Molecular mechanism of the CRISPR-Cas system. When a virus attacks a bacterium, a fragment of the genetic material from the invader will be acquired and integrated as a spacer into the host’s CRISPR locus (1). The CRISPR array is transcribed and further processed into crRNA (2) and upon subsequent attack by the same invader, the spacer will guide the Cas protein to cleave the invading nucleic acid sequence (3), thereby protecting the host.

The CRISPR-Cas system was first discovered in bacteria and later found to confer adaptive immunity against invading bacteriophages and foreign genetic elements [22]. In CRISPR-containing organisms, a molecular memory of an infection is created when fragments of the invading nucleic acid (protospacers) are acquired and integrated as new spacers into the host’s CRISPR locus (Figure 1). A CRISPR locus typically consists of palindromic, short direct repeats of 24–48 nucleotides interspersed by similarly sized, unique spacers that are excised from foreign nucleic acids and the adjacently located CRISPR-associated (Cas) genes. When the CRISPR array is transcribed and processed into mature CRISPR RNAs (crRNAs), the spacer sequence will serve as guide for the Cas protein to specifically recognize and cleave the target nucleic acid, thereby protecting the host from subsequent infection by the same invader [23,24]. The presence of a 2- to 5-nucleotide motif called protospacer-adjacent motif (PAM) in the invading sequence is a prerequisite for the PAM-dependent CRISPR-Cas system to target and cleave foreign nucleic acids while the host genome is protected against self-cleavage by the absence of PAM in the CRISPR locus [25].

The CRISPR-Cas system can be divided into two classes and six types. The two classes differ mainly in the configuration of their effector modules that are involved in crRNA processing and interference. RNA-guided cleavage in a class 1 system (types I, III, and IV) requires a multi-subunit effector complex composed of several Cas proteins, whereas only a monomeric, multi-domain effector protein is involved in a class 2 system (types II, V, and VI) [18]. Due to their relatively simpler organization, class 2 systems have attained widespread adoption as a toolkit for CRISPR-based applications that range from RNA knockdown, editing, imaging, and tracking to nucleic acid detection and regulation of gene expression [26]. In a class 2 type II system, a noncoding trans-activating CRISPR RNA (tracrRNA) is required in addition to the crRNA for Cas9 mediated target cleavage. The tracrRNA and crRNA hybridizes to form a duplex to guide Cas9 to the crRNA-specified target, but the two RNAs can also be synthetically fused to form a single guide RNA (sgRNA) [27]. By customizing the ~20-nucleotide region of the sgRNA that hybridizes with the nucleic acid sequence of interest, specific targeting can be achieved with the Cas9-sgRNA complex to trigger cis-cleavage within the base-pairing region.

Other than cis-cleavage activity, some CRISPR-Cas systems also exhibit sequence-independent nuclease activity that cleaves non-target, single-stranded DNA (ssDNA) or ssRNA in trans. The trans-acting nuclease activity is only activated when the crRNA is bound to an activator through complementary base-pairing. It has been postulated that the target-activated trans-cleavage activity may serve as a coping strategy against phage infection by degrading all RNAs and thus impedes the proliferation of phages or triggers dormancy or suicide as a last resort antiviral response [26,28]. Following the discovery of target-activated trans-cleavage activity in several Cas proteins, the applications of CRISPR-Cas systems for nucleic acid detection have continued to grow with each passing year. Presence of the target activator, which results in the activation of collateral cleavage activity, can be detected using a variety of reporter molecules and this forms the underlying principle of most CRISPR-Cas-based nucleic acid detection platforms [29]. At present, CRISPR-Cas12 has emerged as the most widely applied detection system, followed by CRISPR-Cas13, in the development of CRISPR-based diagnostics (CRISPR-Dx) for COVID-19. Compared to Cas12 and Cas13, the development of Cas3- and Cas9-based detection for the diagnosis of COVID-19 are reported to a lesser extent.

Generally, Cas12 exhibits PAM-dependent cis-cleavage of double-stranded DNA (dsDNA) and PAM-independent cis-cleavage of ssDNA with the trans-cleavage remains only for ssDNA, whereas Cas13 exhibits cis- and trans-cleavage of ssRNA in a PAM-independent manner [30]. On the other hand, Cas3 is only recruited once the target dsDNA flanked by PAM is recognized by the Cas complex for antiviral defense (Cascade) with activation of Cas3 leading to the nicking and degradation of target dsDNA with simultaneous trans-cleavage of non-target ssDNA [31,32]. Cas9, which does not possess trans-cleavage activity, has also been employed for CRISPR-based SARS-CoV-2 detection. Other than utilizing Cas9 for its cis-cleavage activity, the nuclease domains of Cas9 can be mutated to generate a catalytically dead Cas9 (dCas) that lacks nuclease activity but retains its RNA-guided DNA-binding activity [33]. Furthermore, Cas9-sgRNA complexes can be made to target ssRNA for site-specific cleavage in a manner that is similar to PAM-dependent Cas9-mediated dsDNA cleavage by incorporating a DNA-based PAM-presenting oligonucleotide (PAMmer) that binds to the targeted ssRNA [34]. A comparison of major characteristics of the Cas proteins used for CRISPR-based SARS-CoV-2 detection is presented in Table 1, including their targeting requirements (such as PAM and protospacer flanking sequence (PFS) and guide RNA requirements), cis- and trans-cleavage activities, and on- and off-target substrates.

## 3. An Overview of CRISPR-Dx Workflow

**Figure 2:**
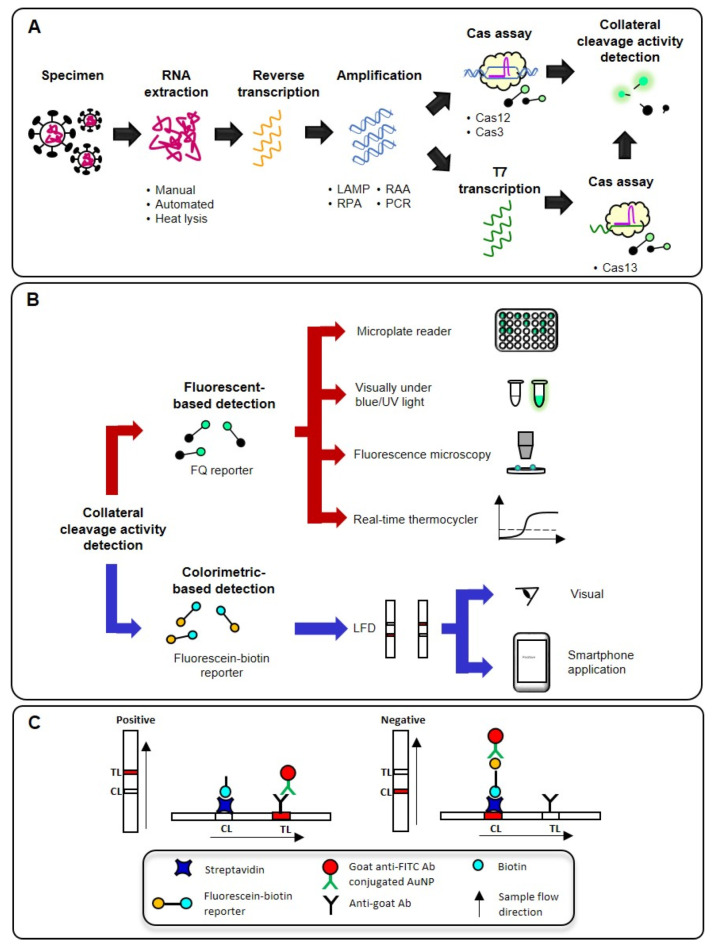
(**A**) Typical workflow of various CRISPR-Dx for COVID-19 starting from RNA extraction, reverse transcription, amplification, Cas assay, and detection of collateral cleavage activity. (**B**) Various strategies for the detection of collateral cleavage activity which can be divided into fluorescent-based and colorimetric-based detection. (**C**) Detection of fluorescein-biotin reporter following Cas assay with a LFD in which the reporter is either cleaved in a positive reaction or remains intact in a negative reaction. Ab: antibody; AuNP: gold nanoparticles; CL: control line; LAMP: loop-mediated isothermal amplification; RAA: recombinase-aided amplification; RPA; recombinase polymerase amplification; TL: test line.

The typical workflow of a CRISPR-Dx for COVID-19 consists of RNA extraction, reverse transcription (RT), target amplification, Cas assay, and collateral cleavage activity detection as shown in Figure 2A. RNA extraction is firstly carried out to lyse and purify the RNA genome of SARS-CoV-2 from clinical specimens, such as nasopharyngeal swab [35,36,37,38,39] nasal swab [40], oropharyngeal swab [14,41], saliva [42,43], bronchoalveolar lavage [35,39] and sputum [35]. The viral RNA is then converted into complementary DNA through RT followed by a DNA-based amplification technique in a one-step or a two-step approach to generate a large amount of target DNA prior to the Cas assay and collateral cleavage activity detection. The amplification step is generally required because the low amount of target sequence in a clinical specimen is undetectable by the Cas protein [35,44]. The N gene of SARS-CoV-2 is the most common target (63%) for CRISPR-Dx followed by Orf1ab (28%), E (23%), S (12%), RdRp (5%), and Orf8a (5%). In the case of Cas13, which recognizes RNA as the on-target substrate instead of DNA, an additional step of converting the amplified DNA into RNA via T7 transcription will be needed to activate the collateral cleavage activity of Cas13. By incorporating reporter molecules as the off-target substrates, various detection methods ranging from low-throughput, instrument-free to high-throughput, instrument-dependent ones can be used based on the application contexts (Figure 2B).

Nucleic acids are most commonly amplified via the PCR process, but a specialized thermal cycling instrument is required and integration of the thermocycler with an optical system for real-time PCR applications further increases the upfront cost, making PCR-based diagnostics costly and inappropriate for resource-limited, field, or POC settings. Isothermal amplification techniques such as LAMP, RPA, and RAA have simpler instrument requirement because amplification of the target sequence occurs at a constant temperature which can be easily achieved using a water bath or a heat block. A typical LAMP reaction can be completed within an hour to produce more than 10^9^ copies of target gene. However, unlike PCR, LAMP requires a DNA polymerase with strand-displacement activity and uses at least four primers to target six distinct regions of the target sequence [45]. On the other hand, RPA and RAA are two-primer-based isothermal amplification methods that require the presence of three core enzymes (recombinase, ssDNA binding protein, and strand-displacing DNA polymerase) [46]. The UvsX recombinase used in RPA is derived from T4 phage, whereas that used in RAA is derived from *Escherichia coli*. The lower amplification temperatures of RPA and RAA (37–42 °C) as compared to LAMP (60–65 °C) also paved the way for the development of a one-pot assay that combines target amplification and Cas assay in a single tube. Although isothermal amplification techniques are highly sensitive, false positive results could still occur due to non-specific amplification and thus, there is a need to couple these amplification techniques with a sequence-specific detection method such as the CRISPR-Cas system [38].

The collateral cleavage activity of the Cas protein that is activated by the presence of the target sequence can be detected fluorometrically by utilizing a fluorescent-quencher (FQ) reporter. An intact FQ reporter does not fluoresce due to the close proximity between the fluorophore and quencher, but cleavage of the FQ reporter will release the fluorophore, allowing the fluorescent signal emitted to be detected under blue or UV light by various methods, including by the naked eye, fluorescent microscopy, a plate reader, and even a real-time thermocycler (Figure 2B). Alternatively, the reporter may be labeled with biotin and fluorescein at opposing ends for detection on a lateral flow device (LFD) such as the Millenia HybriDetect strip (TwistDx, Cambridge, UK) (Figure 2C). When a negative sample is applied onto the sample pad of the strip, goat anti-fluorescein antibody-conjugated gold nanoparticles (AuNPs) deposited on the conjugate pad will bind to the fluorescein label of the intact reporters. Accumulation of the AuNP-bound intact reporters, which are captured and immobilized by streptavidin, will result in the appearance of a purplish-red colored control line. If the reporters are cleaved by the Cas protein, a purplish-red colored, anti-goat antibody-coated test line will appear instead due to the capture and immobilization of goat anti-fluorescein antibody-conjugated AuNPs. This allows the LFD result to be read by the naked eye or the line intensity could be measured with an image processing program. In the following sections, we will present the latest developments and explorations of the CRISPR-Cas system in the field of COVID-19 diagnostics with a primary focus on the assay designs and performance characteristics associated with this technology. Detailed characteristics of these CRISPR-Dx for SARS-CoV-2 detection are summarized in Table 2.

## 4. Cas12-Based CRISPR-Dx

### 4.1. Two-Pot Assays

Given that rRT-PCR is deemed as the standard diagnostic test for the confirmation of COVID-19, Huang et al. [41] demonstrated the ease of coupling RT-PCR with a CRISPR-Cas12a assay, termed specific enhancer for detection of PCR-amplified nucleic acids (SENA), to improve the sensitivity and specificity of the technique for SARS-CoV-2 detection [41]. Following the completion of the RT-PCR reaction, the SENA reaction is set up by adding the amplicons to the SENA reagent (LbCas12a, crRNAs, and FQ reporter) and the increase in fluorescent signal due to the cleavage of the FQ reporter is then measured with a fluorescence reader. By using a mixture of crRNAs targeting the Orf1ab and N genes, the authors found that the mix-SENA was more sensitive than detecting each of the targets alone and showed that the limit of detection (LoD) of mix-SENA (1.6 copies/reaction) was lower than that of rRT-PCR (4.0 copies/reaction). As such, mix-SENA may play a role in resolving samples with ambiguous rRT-PCR results that are associated with high cycle threshold (Ct) value “grey zones”. Mix-SENA was also able to identify two false positives and four false negative results by rRT-PCR as corroborated by next-generation sequencing results when evaluated with 295 clinical specimens. The potential application of mix-SENA as an indicator of viral clearance was also demonstrated with samples from three COVID-19 recovering patients, whereby rRT-PCR-negative samples were found to be positive by mix-SENA, highlighting the risk of patients being discharged prior to complete viral clearance [41].

A particular CRISPR-Cas12 detection system may also be developed to be compatible with both non-isothermal- and isothermal-based amplification techniques. For example, the CRISPR-based fluorescent diagnosis system for COVID-19 (COVID-19 CRISPR-FDS) developed by Huang et al. [40] could be used to detect RT-PCR- or RT-RPA-amplified N and Orf1ab genes without changes in the detection limit of the test [33]. Furthermore, the LoD of the COVID-19 CRISPR-FDS (2 copies/test) was reported to be comparable to that of rRT-PCR (5 copies/test). Based on the analysis of 29 nasal swab specimens from suspected COVID-19 cases, CRISPR-FDS showed complete concordance with the state laboratory-generated rRT-PCR positive samples (100% PPA), but not with rRT-PCR negative samples (71.4% NPA). The authors could not conclude whether the three discordant samples represented false positive CRISPR-FDS or false negative rRT-PCR results due to the lack of information and further testing. The large discrepancy between the rRT-PCR results of the 29 nasal swab specimens generated by a hospital laboratory and the state laboratory in the study further emphasizes the need for diagnostic tests that are not only rapid and sensitive, but also robust in detecting SARS-CoV-2 positive samples [40].

In terms of target amplification, isothermal amplification-based CRISPR-Cas assay is the preferred approach for COVID-19 diagnosis with DNA endonuclease-targeted CRISPR trans reporter (DETECTR) being a typical representative of the Cas12-based detection schemes. Notably, the SARS-CoV-2 DETECTR Assay and the SARS-CoV-2 DETECTR Reagent Kit are the first and only CRISPR-Cas12-based diagnostic tests to receive an emergency use authorization (EUA) from the United States Food and Drug Administration (FDA) in July and August 2020, respectively [78]. The assay consists of two monoplex reactions and is designed to amplify the target N gene and internal control RNase P separately. RNA extraction is a prerequisite, and the RNA extract serves as a template for the 30-min RT-LAMP reaction at 62 °C followed by a 15-min Cas12 assay at 37 °C. A real-time thermocycler is required for fluorescence measurement and a cut-off value of 500,000 relative fluorescent units is used to interpret positive/negative result for the target and control. The SARS-CoV-2 RNA DETECTR Assay [79] and SARS-CoV-2 DETECTR Reagent Kit [47] share the same performance characteristics (LoD = 20 copies/µL; PPA = 95%; NPA = 100%), but the test is only authorized to be conducted in Clinical Laboratory Improvement Amendments (CLIA)-certified laboratories that meet the requirements to perform high complexity tests. Despite similar personnel and instrument requirements, the SARS-CoV-2 DETECTR Assay was six- to twenty-fold less sensitive than the FDA-EUA approved CDC 2019 novel coronavirus (2019-nCoV) real-time RT-PCR diagnostic panel (1–3.16 copies/µL) [80].

In the RT-LAMP-DETECTR assay developed by Broughton et al. [14], the E gene was used to detect three SARS-like CoVs (SL-CoVs), including SARS-CoV-2, bat SL-CoV, and SARS-Cov, whereas the N gene was used to detect SARS-CoV-2 specifically. The target genes, along with an internal control RNase P, were amplified individually in separate tubes prior to CRISPR-Cas12a detection [14]. Other than Broughton et al. [14], many researchers have also sought to demonstrate the feasibility of using either an LFD or a fluorescence reader for CRISPR-Cas result interpretation without compromising the test performance [35,48]. This flexibility provides the test operator with the option of using either one of the devices based on the resources that are available as well as the expected throughput and turnaround time in a particular setting. Other researchers opted for naked eye detection of fluorescence emission of positive samples under blue or UV light, such as in the RT-RPA-coupled CRISPR-Cas12a assays developed by Wang et al. [49] and Mayuramart et al. [50]. Although results interpretation based on naked eye detection is advantageous for POC and resource-limited settings, the risk of operator bias cannot be avoided due to the subjective nature of visual inspection.

Other than LFD and fluorescence reader, a low-cost, three-dimensional (3D)-printed, smartphone-based device for fluorescence imaging has been described for the analysis of RT-RPA-DETECTR results [36]. The highly portable device consists of a sample tray with a capacity of eight PCR tubes, a smartphone holder, and an imaging compartment. Images captured with the phone camera are then sent to the cloud where a machine learning algorithm will be used for result interpretation. The LoD of this RT-RPA-DETECTR assay (6.25 copies/µL) was comparable to the RT-LAMP-DETECTR assay (10 copies/µL) developed by Broughtonet al. [14], but higher than that of rRT-PCR (1–3.2 copies/μL) [14,36,80]. The PPA and NPA of the RT-LAMP-DETECTR assay relative to rRT-PCR (*n* = 82; PPA = 95%; NPA = 100%) [14] were also better than the RT-RPA-DETECTR assay (*n* = 115; PPA = 87%; NPA = 92%) [36]. Nevertheless, the PPA and NPA of this RT-RPA-DETECTR assay improved to 97% and 93%, respectively, when clinical samples were restricted to those with high viral loads (Ct < 33; *n* = 96), indicating that the developed model required further fine-tuning on clinical samples with low viral loads [36].

### 4.2. One-Pot Assays

Post-amplification analysis that requires the opening and closing of the amplification reaction tube, particularly the isothermal amplification reaction tube, increases the risk of aerosol contamination that can lead to false positive results. Therefore, the development of a closed-tube format for CRISPR-Dx would be ideal to minimize the risk of carry-over and cross contaminations. The RT-All-In-One Dual CRISPR-Cas12a (RT-AIOD-CRISPR) assay is one of such examples whereby RT-RPA and CRISPR-Cas12a reagents are added in a single tube and incubated at 37 °C for 40 min before the result is visualized under blue or UV light [52]. Unlike the DETECTR assay in which the collateral activity of Cas12 is induced by the binding of crRNA to dsDNA in a PAM-dependent manner, RT-AIOD-CRISPR relies on crRNAs that are designed without PAM so that the binding of crRNAs to single-stranded amplicon during RPA will still induce the collateral cleavage activity of Cas12. In addition to achieving a LoD of 5 target RNA copies and complete concordance with rRT-PCR results when tested with 28 clinical samples, the authors also showed that the RT-AIOD-CRISPR assay could be performed with a hand warmer and positive results could be observed in as little as 20 min [52].

Contrary to the strategy used by Ding et al. [52], other researchers sought to avoid the cis-cleavage activity of Cas12 during the amplification process physically by separating the CRISPR-Cas reaction mixture from the amplification reaction mixture within the confine of a single tube. This is typically achieved by placing the CRISPR-Cas reaction mixture within the lid of the tube while the amplification reaction mixture is placed at the bottom of the tube with or without a layer of mineral oil [53,54,55,56,57]. Upon completion of the amplification process, the solution is either mixed by inverting the tube manually or subjecting the tube to a brief spin. Due to the use of RT-LAMP as the amplification method, the assay protocol developed by Chen et al. [53], Wanget al. [54], and Pang et al. [55] required different incubation temperatures for amplification and Cas12, assay whereas the RT-RPA-based OR-DETECTR assay developed by Sun et al. [56] only requires a single incubation temperature. Result are then interpreted based on visual inspection under blue/UV light or via a fluorescence read-out. The reported LoD for these one-pot assays ranged from 2.5 copies/µL to 45 copies/µL and achieved 97%–100% concordance with rRT-PCR results when tested with clinical specimens (*n* = 14–100) [54,55,56]. Like Samacoits et al. [36], Chen et al. [53] also capitalized on 3D printing technology to fabricate a portable instrument for fluorescence imaging with a smartphone camera, but result interpretation was based on visual inspection instead of a cloud-based analysis and the LoD attained was 20 copies/reaction [53].

As RT-LAMP-based CRISPR-Cas12a detection requires different incubation temperatures, this drawback can be overcome by substituting Cas12 with a thermostable ortholog such as the Cas12b from *Alicyclobacillus acidiphilus* (AapCas12b) and *Alicyclobacillus acidoterrestris* (AacCas12b). Unlike LbCas12a, which operates at an optimal temperature of 37 °C, AapCas12b is able to function at temperatures up to 65 °C [37], making it compatible with RT-LAMP to create CRISPR-Cas12b-based one-pot assays that only require a single incubation temperature. For example, the in vitro specific CRISPR-based assay for nucleic acids detection (iSCAN) developed by Ali et al. [51] began as a two-pot assay in which RT-LAMP (62 °C, 30 min) and Cas12a assay (37 °C, 10 min) were performed in separate tubes [51]. To further simplify the assay protocol, the team proceeded to develop a one-pot iSCAN by replacing LbCas12a with the thermophilic variant AapCas12b. When the RT-LAMP and Cas12b reagents were added together, lower amplification efficiency was achieved as compared to the two-pot format. This was attributed to the cleavage of target amplicon by the activated Cas12b during the amplification process. Hence, the CRISPR-Cas12b reagent mixture was placed on the tube wall near the top of the tube to allow the RT-LAMP reaction (62 °C, 30 min) to proceed to completion. The tube was then subjected to a brief spin followed by the Cas12b assay (62 °C, 15 min) and detection. The one-pot and two-pot iSCAN exhibited the same LoD (10 copies/reaction) and were two-fold higher than that of rRT-PCR (5 copies/reaction). Evaluation with 24 clinical specimens revealed that the PPA and NPA of the one-pot and two-pot iSCAN using fluorescent-based detection were the same. Specifically, the PPA of iSCAN was dependent on the target gene (N gene, 85.7%; E gene, 38.1%), whereas NPA was 100% for both formats [51].

In the SHERLOCK Testing in One Pot (STOP) SARS-CoV-2 (STOPCovid.v2) assay [37], a 10-min magnetic bead-based RNA extraction was first performed and, by retaining only the RNA-bound magnetic beads in the tube under a magnetic field, the same tube was used for the RT-LAMP and Cas12 assay by adding the STOPCovid.v2 reaction mixture to the beads. The tube was then incubated at 60 °C in a real-time thermocycler for 1–2 h with fluorescence measurements taken 1 h prior to LFD-based detection. Compared to the LoD of rRT-PCR (1000 copies/mL), the LoD of STOPCovid.v2 (33–83 copies/mL) was found to be 12–30 times lower. Evaluation of the STOPCovid.v2 with 402 clinical samples yielded a PPA of 93.1% and an NPA of 98.5% [37]. Guo et al. [57] coupled RT-RAA with a CRISPR-Cas12b-mediated DNA detection (CDetection) to develop a CRISPR-assisted detection (CASdetec) platform [57]. Due to the drastic decrease in sensitivity when RT-RAA and CDetection were concurrently executed within a single tube, Guo and colleagues separated the RT-RAA (42 °C, 30 min) and CDetection (42 °C, 30 min) reaction mixtures by placing the CDetection reagents within the lid of the tube. A brief spin was sufficient to bring the CDetection reagents down after RT-RAA was completed. Measurement of the fluorescence emission with a fluorescence reader resulted in a LoD of 1 × 10^4^ copies/mL of SARS-CoV-2 pseudovirus. Despite the apparent advantage of using AapCas12b due to its thermostable nature, the longer sgRNA required as compared to the crRNA of LbCas12a may increase the risk sporadic collateral activity arising from the overlapping between the sgRNA and LAMP primers [55].

### 4.3. Other Assay Formats

The work of Ramachandran et al. [58], Park et al. [59], and Ning et al. [42] highlights the use of a chip-based approach that consumes less reagents than conventional devices. Ramachandran et al. [58] used an electrokinetic microfluidic technique called isotachophoresis (ITP) to automate the RNA extraction process and to control the Cas assay within an in-house built microfluidic chip through the application of an electric field. The major disadvantage of the present ITP-CRISPR design for SARS-CoV-2 detection is the off-chip steps whereby sample lysis and RT-LAMP remain as tube-based procedures. The ITP-CRISPR in its current design also requires laboratory-based instruments (such as a source meter and camera-mounted inverted epifluorescence microscope) and the LoD obtained (10 copies/µL) was similar to that achieved with LFD in other CRISPR assays [14,48,56]. Nonetheless, the sample-to-result time of ITP-CRISPR (~35 min), which is inclusive of the RNA extraction step, is still shorter than RNA extraction-free CRISPR-based assays (50–75 min) [60,61,62]. Furthermore, ITP-CRISPR is amenable to automation, miniaturization, and integration of different analytical operations. The development of its associated detection systems into hand-held devices would make the platform applicable for POC use.

Park et al. [59] took a different approach and utilized a commercially available chip (QuantStudio 3D Digital PCR 20K Chip, Thermo Fisher Scientific) to develop a digital CRISPR-Cas-based assay called digitization-enhanced CRISPR/Cas-associated one-pot virus detection (deCOViD) [59]. Both ITP-CRISPR and deCOViD are designed for monoplex detection of target gene with a single sample loaded per chip, but unlike ITP-CRISPR [58], the only off-chip step in deCOViD is the sample processing step in which either RNA extract or heat-inactivated SARS-CoV-2 is obtained. Once the template is added to the mixture of reagents for RT-RPA and Cas12a assay, a specialized chip loader will be used to partition the mixture into 20,000 nanoscale reaction wells that are etched on the chip. Subsequently, deCOViD required only a single incubation step at 42 °C for 30 min, which is carried out in a custom-assembled miniature heater, before fluorescence intensity is measured under a fluorescence microscope [59]. A five- to ten-fold increase in sensitivity was observed when the LoD of deCOViD (20 GE/µL heat-inactivated SARS-CoV-2; 1 GE/µL) was compared to that of RT-RPA-CRISPR detection with a real-time thermocycler (100 GE/µL heat-inactivated SARS-CoV-2; 10 GE/µL RNA). Although deCOViD was shown to accelerate qualitative and quantitative detection along with a broad dynamic range and increased sensitivity through digitization, its highly specialized equipment requirement (such as a chip loader and fluorescent microscope) would need to be addressed if the platform were to gain acceptance for more widespread use.

A 15-min sample-to-result, chip-based assay that combines RT-RPA, CRISPR-Cas12a, and a fluorescence detection system (FDS) was recently described by Ning et al. [42], making this proof-of-concept study a breakthrough in CRISPR-Dx for COVID-19. The CRISPR-FDS assay, which is designed to analyze saliva samples following a 5-min lysis step, utilizes a compact, in-house built chip containing five reaction wells that can accommodate the analysis of five assays in parallel with a smartphone-based fluorescence microscope [42]. To conduct the assay, an aliquot of lysed sample is added to the reaction well of a chip that is pre-filled with premixed RPA and CRISPR-Cas solution. The chip only requires a 10-min incubation step at room temperature before it is ready to be inserted into a smartphone-based fluorescence microscope for imaging under blue light. The CRISPR-FDS assay developed by Ning et al. [42] demonstrated good linearity over a broad range of viral concentrations (1–10^5^ copies/µL) with a calculated LoD (0.38 copies/µL) below that of the CDC 2019 novel coronavirus (2019-nCoV) real-time RT-PCR diagnostic panel (1–3.16 copies/µL). A clinical evaluation with 103 saliva and 103 nasal swab samples also revealed that the performance of CRISPR-FDS in relation to rRT-PCR was similar (PPA = 99%; NPA = 99%) when using either the smartphone-based fluorescence microscope or a plate reader. Furthermore, viral load was also found to be correlated in the 43 saliva samples that were CRISPR-FDS- and rRT-PCR-positive (r = 0.63). Nonetheless, further improvements that include on-chip sample lysis, incorporation of microfluidic channels, and the development of a custom smartphone app for assay regulation and result analysis have been proposed to make the platform more user-friendly for POC testing [42].

Wu et al. [60] demonstrated how a low-cost polypropylene (PP) bag-based approach may be used to facilitate at-home COVID-19 nucleic acid testing [60]. The three-chamber PP bag was designed to be flexible so that mixing could be performed by pressing the chamber with fingers and a foam is also placed in the lid of the PP bag to enable the device to float on water. More importantly, the PP bag allowed simultaneous amplification of the target gene (Orf gene) and an internal control RNase P as well as the subsequent CRISPR-Cas detection to be conducted in a closed environment. To perform the assay, lysis buffer, wash buffer, and RT-LAMP reagents are placed into the respective chamber within the PP bag before a layer of oil is added to form an immiscible interface between the different chambers. Magnetic particles followed by a sample is then added into the lysis chamber and the solution is mixed before the PP bag is incubated at 65 °C for 10 min in a milk warmer. An external magnet is used to transfer the RNA-bound magnetic particles between chambers. Adsorption of the RNA on magnetic particles allows the nucleic acids to be transferred to the wash chamber and finally to the amplification chamber where RT-LAMP takes place at 65 °C for 30 min. The CRISPR-Cas reagent mixture for the target gene is only added at the end of the amplification process and a further incubation at 37 °C for 10 min is required prior to the visualization of fluorescence emission with a portable UV lamp in a dark room. A visible fluorescent signal indicates that the sample is positive, but a negative sample must be verified with a second round of CRISPR-Cas assay for RNase P to rule out a false negative result [60]. The LoD obtained (20 copies/reaction) was the same as that achieved in the tube format with a real-time thermocycler as well as that of the visual-based, one-pot RT-LAMP-CRISPR assay developed by Chen et al. [53].

### 4.4. RNA Extraction-Free Protocols

Development of CRISPR-Dx with the capability to detect SARS-CoV-2 from unpurified clinical specimens is of particular interest to researchers due to the reduction in sample-to-result time, the number of liquid-handling steps, and reliance on laboratory-based equipment (such as a centrifuge). Manual RNA extraction involves multiple liquid-handling steps and hence, is tedious and time-consuming particularly when dealing with a large number of samples. Automated RNA extraction instruments provide a walk-away solution and free up personnel for other tasks, but such instruments are expensive, making them useful and cost effective if used in large laboratories with high-throughput testing. For example, the Qiagen EZ1 DSP Virus Kit (Qiagen, Hilden, Germany) that was validated for RNA extraction in the FDA-EUA approved SARS-CoV-2 DETECTR Reagent Kit, has an estimated time per run of 40 min on the EZI Advanced XL instrument (Qiagen, Hilden, Germany). Therefore, it would be ideal, particularly for POC testing, if the RNA extraction could be substituted with a simple specimen processing method such as a 5-min heat lysis step at 80 °C, as described by Xiong et al. [61], that was shown to be capable of liberating SARS-CoV-2 genomic RNA for RT-RPA [61]. Based on visual inspection of the LFD, analytical sensitivity of the RT-RPA-CRISPR-Cas12 assay (1 copy/μL) was 10–50 times more sensitive than RT-RPA alone (10-50 copies/μL) [61]. Similarly, Garcia-Venzor et al. [62] showed that a 25-min heat lysis protocol (42 °C for 20 min; 64 °C for 5 min) with a lysis buffer could be used to generate RNA template for a RT-LAMP-CRISPR-Cas12a assay and measurement of fluorescence with a real-time thermocycler resulted in a LoD of 16 copies/µL [62]. Other RNA extraction-free methods that have been described include a 5-min lysis step at 37 °C [42], a 5-min proteinase K and heat treatment at 95 °C [63], a 30-min heat inactivation step at 65 °C [59], and a 10-min magnetic bead-based purification step [37]. These simplified protocols highlight the potential use of unextracted samples for isothermal amplification techniques to achieve significant reduction in the total assay time of CRISPR-Cas12a-based diagnostics.

### 4.5. Sensitivity and Specificity Enhancement Strategies

In an attempt to enhance the sensitivity and specificity of CRISPR-Cas assays as well as to minimize mutational escape, several researchers have chosen to use multiple guide RNAs that bind to different regions of the target gene [35,49,50,52,63,64]. Moreover, the activation of more Cas12a protein per unit time by multiple crRNAs would also lead to greater signal amplification as compared to that of single crRNA, leading to higher sensitivity [64]. In the RT-LAMP-DETECTR assay developed by Brandsma et al. [35], two guide RNAs were used to target different regions of the N gene; the analytical sensitivity of the resulting assay was found to be 10–100 times more sensitive than rRT-PCR when tested with 10-fold dilutions of RNA extract from four COVID-19 patients [35]. Cross-reactivity was not observed with four other human CoVs, suggesting an analytical specificity of 100%. The authors also demonstrated that Cas12 ribonucleoprotein (RNP) was incapable of detecting SARS-CoV-2 RNA following RT even in samples with high viral load (Ct < 20), whereas the combination of RT-LAMP and Cas12 resulted in an assay that exhibited higher sensitivity than either of the methods alone. Evaluation of this RT-LAMP-DETECTR assay with a large cohort of COVID-19 patients (*n* = 378) from three hospitals showed a 94.9% concordance with rRT-PCR results [35].

Another strategy that may lead to the universal enhancement of CRISPR-Cas12a system is to use engineered crRNA [63,65,81]. By combining the CRISPR-enhanced analysis of nucleic acids with CrRNA extensions (ENHANCE) technology with lateral flow-based detection of the RT-LAMP-amplified N gene, Nguyen et al. [65] showed that the band intensity obtained with engineered crRNA containing a 7-mer AT-rich 3′-overhang was stronger due to the augmentation of Cas12a-mediated collateral cleavage activity as compared to that of un-engineered crRNA [65]. In the S gene-targeting variant nucleotide guard (VaNGuard) assay developed by Ooi et al. [63], the authors not only capitalized on the enhancement brought about by using engineered crRNAs, but also that of an engineered variant of AsCas12a (enAsCas12a) [63]. Compared to LbCas12a, the enAsCas12a was found to be active over a wide range of temperatures (37 °C to 65 °C) and exhibited a higher mismatch tolerance that makes it robust against viral genome mutations. Ooi and colleagues also explored the effect of different crRNA modifications on the collateral activity of enAsCas12a, including 3′- or 5′-extended crRNAs, crRNAs bearing 2′-O-methyl ribonucleotides, 2′-deoxy-2′-fluoro-ribonucleotides, and phosphorothioate linkages as well as DNA–RNA hybrid crRNAs. Using unmodified crRNAs as the benchmark, the DNA–RNA hybrid crRNAs were able to increase the rate of enAsCas12a detection reaction at 60 °C while suppressing collateral activity in the absence of the target sequence to negligible levels [63]. In another study, Ma et al. [64] discovered that manganese ion could increase the sensitivity of Cas12a detection up to 13-fold after screening a series of divalent cations [64]. The manganese-enhanced Cas12a (MeCas12a) system was then developed to detect the RT-RAA-amplified E gene of SARS-CoV-2, but an additional step of desalting the amplicon was required prior to the MeCas12a assay. The MeCas12a assay exhibited a LoD of 5 RNA copies and perfect agreement with rRT-PCR results when evaluated with 24 clinical nasopharyngeal specimens [64].

## 5. Cas13-Based CRISPR-Dx

### 5.1. Two-Pot Assays

Many CRISPR-Cas13-based detections of SARS-CoV-2 described to date consist of a nucleic acid amplification step, during which a T7 RNA polymerase promoter is incorporated into the amplicons, followed by simultaneous T7 transcription and Cas13a (LwaCas13a) detection via a fluorescence reader or LFD [38,39,66,67]. Most of these tests were built on the specific high-sensitivity enzymatic reporter unlocking (SHERLOCK) technology and in fact, the Sherlock CRISPR SARS-CoV-2 Kit is the first CRISPR-Dx for COVID-19 to receive FDA-EUA in May 2020 [78]. The Sherlock CRISPR SARS-CoV-2 kit is a monoplex-based assay that targets the Orf1ab and N genes with RNase P serving as an internal control. Using RNA extract as template, a 40-min RT-LAMP reaction is carried out to amplify the target sequence while simultaneously embedding a T7 polymerase promoter sequence into the amplicons. A further 10-min incubation at 37 °C for transcription and Cas13 assay takes place in a plate reader that also measures the fluorescent signal at 2.5-min intervals. A minimum of a 5-fold increase in fluorescence measurement over the corresponding non-template control at minute 10 is used to denote a positive reaction. Overall, the Sherlock CRISPR SARS-CoV-2 kit (LoD = 6.75 copies/µL; PPA = 100%; NPA = 100%) showed better performance than that of the FDA-EUA approved SARS-CoV-2 DETECTR Reagent Kit (LoD = 20 copies/µL; PPA = 95%; NPA = 100%). In terms of assay reagents, the SARS-CoV-2 DETECTR Reagent Kit comes with user-friendly, pre-prepared DETECTR master mixes, whereas the CRISPR-Cas master mixes in the Sherlock CRISPR SARS-CoV-2 kit have to be prepared manually from multiple components. A drawback that is shared by both the Sherlock CRISPR SARS-CoV-2 Kit and SARS-CoV-2 DETECTR Reagent Kit is the monoplex format employed, because it increases the number of liquid handling steps as well as sample and reagent consumption in comparison to multiplexed real-time rRT-PCR tests.

In subsequent research by Patchsung et al. [38], a SHERLOCK assay targeting the S gene of SARS-CoV-2 with a LoD of 42 copies/reaction was firstly evaluated with 154 clinical samples [38]. The PPA value was found to be higher when fluorescent readout was used (96%) as compared to that of LFD (88%), although both methods showed 100% NPA. Due to the higher sensitivity of fluorescent readout, Patchsung and colleagues also investigated the use of a blue light to visualize the SHERLOCK results of 380 pre-operative patients and found that the results were in complete concordance with those of rRT-PCR. While RNase P is commonly used as a nucleic acid extraction procedural control and to rule out false negative results, Patchsung et al. [38] elected to use an RNA reporter instead as an internal control to detect RNase contamination. RNase can severely impact the performance of CRISPR-Cas13-based assays because degradation of RNA templates can lead to false negative results, whereas cleavage of RNA reporters due to RNase contamination can lead to false positive results. To carry out its function as an internal control, the RNA reporter incorporated into the SHERLOCK assay has been designed to be resistant to LwaCas13a cleavage but not RNase I, RNase A, and RNase T1 cleavage. By utilizing different haptens as labels, the RNA reporters for the target (biotin) and internal control (digoxigenin) could be separately captured and differentiated with an LFD [38].

Rauch et al. [68] attempted to address some of the limitations surrounding nucleic acid amplification testing by developing Cas13-based, rugged, equitable, scalable testing (CREST) that pairs RT-PCR–CRISPR-Cas13a with low-cost, portable instrumentation without compromising the sensitivity of the test [68]. Instead of using an isothermal amplification technique that entails the use of reagents that are supplied by a small number of manufacturers, Rauch et al. [68] opted for PCR that utilizes the commonly available Taq polymerase for target amplification. Thermal cycling was conducted in a battery-operated, mini-PCR mini16 (miniPCR bio, Cambridge, MA, USA) that can be connected to a smartphone. Following transcription and Cas13 assay, fluorescence emission was visualized with a battery-powered P51 Molecular Fluorescent Viewer (miniPCR bio, Cambridge, MA, USA) that houses a blue light. As compared to a one-step TaqMan rRT-PCR assay, CREST was not only found to have a similar LoD (10 copies/µL) but also reduced the upfront instrumentation cost by 30 to 50-fold and lowered the total cost of reagents. An evaluation with 218 clinical samples showed that CREST has a PPA and NPA of 97% and 98%, respectively [68].

### 5.2. Label-Free Assay

An innovative approach in the form of a label-free CRISPR-Cas13a-based transcription amplification method with the ability to detect and differentiate SARS-CoV-2 variants in the single nucleotide range was reported by Wang et al. [69]. A set of probes with an embedded promoter sequence is used to bind specifically to the target RNA and a template for transcription is created when the probes are ligated by T4 RNA ligase 2. By utilizing a ligation-based transcription amplification method, the use of reverse transcriptase that are inherently error prone and the need for thermal cycling can be circumvented. Instead of the conventional FQ reporters, Wang et al. used a light-up RNA aptamer as the reporter molecule because formation of the RNA aptamer-DFHBI-1T complex enhances the fluorescence of DFHBI-1T by 430 times. Activation of the Cas13 in the presence of the target sequence would then result in a significant reduction in fluorescence as measured with a fluorescence reader due to the substantial cleavage of the RNA aptamer. Potential of the assay for single nucleotide variation detection was also shown as the D614G mutation in the spike protein of SARS-CoV-2 could be successfully distinguished from its wild-type counterpart. The assay has a LoD of 82 copies/reaction and a preliminary evaluation demonstrated the ability of the assay to detect SARS-CoV-2 in samples that are spiked with SARS-CoV-2 pseudovirus (throat swabs, food packaging, and frozen belt fish samples) and SARS-CoV-2-positive clinical samples (throat swabs) [69].

### 5.3. Amplification-Free Assay

Fozouni et al. [70] took a different approach from that of Rauch et al. [68] by developing an amplification-free, CRISPR-Cas13a assay that uses a portable smartphone-based microscopy for fluorescence measurement [70]. The assay is also a breakthrough in CRISPR-Dx for COVID-19 as it allows the direct quantification of viral load, providing clinicians with valuable information that aids in the prediction of disease progression, infectivity, recovery, and return from quarantine. The assay involved the use of multiple crRNAs in combination to target the N and E genes of SARS-CoV-2 as well as LbuCas13a from *Leptotrichia buccalis* due to its higher sensitivity and robust collateral cleavage activity as compared to other Cas13a homologs. The assay is performed by mixing the extracted RNA sample with Cas13a reagents before the mixture is loaded into a three-channel sample chip. The chip is then incubated at 37 °C within the smartphone-based device comprising a fluorescence microscope and a reaction chamber. A smartphone is placed on top of the device to enable the camera to capture the fluorescence signal generated by Cas13a at a predetermined time interval [70]. Compared to CREST, the Cas13a exhibited lower sensitivity as the LoD was 10-fold higher (~100 copies/µL). Of note is the assay described by Rauch et al. [68] and Fozouni et al. [70], which demonstrated how some, but not all, of the specialized laboratory equipment used could be replaced with portable, low-cost alternatives. Therefore, further work will be required to realize the full potential of both assays for field deployment, such as substitution of the lab-based RNA extraction step with a simplified sample processing protocol, optimization of the long-term enzyme storage conditions, and a reduction in the number of liquid handling steps to facilitate POC testing.

### 5.4. Strategies for High-Throughput Analysis

The specificity of CRISPR-Cas13a could also be harnessed for highly multiplex nucleic acid detection as exemplified by the combinatorial arrayed reactions for multiplex evaluation of nucleic acids (CARMEN)-Cas13 assay that can simultaneously differentiate 169 human viral pathogens, including SARS-CoV-2 [67]. The high-throughput capacity of CARMEN-Cas13 is made possible by the development of 1050 color codes and a massive-capacity chip (mChip) with 177,000 wells. The mChip allows more than 4500 replicated tests to be run per chip while reducing the cost of reagent by more than 300-fold as compared to multiwell-plate SHERLOCK assay. To perform the CARMEN-Cas13 assay, each PCR- or RPA-amplified sample and Cas-13 detection mixture containing Cas13, a crRNA, and an FQ reporter is first combined with a distinct fluorescent color code that will act as an optical identifier. The color-coded solutions are then emulsified into nanoliter droplets, pooled, and loaded into an mChip. As each microwell accommodates two droplets from the pool at random, fluorescence microscopy is used to determine the contents as well as to monitor the detection reaction in each microwell for up to 3 h. The imaging data are subsequently analyzed with in-house, customized Python scripts. Nevertheless, the clinical utility of CARMEN-Cas13 in detecting SARS-CoV-2 has yet to be demonstrated as only dengue, Zika, influenza A, HIV, and HCV patients’ samples were tested and compared with next-generation sequencing results [67]. Due to the highly multiplexed nature of the assay, there were more manual liquid-handling steps involved and a higher upfront cost is also required due to the use of specialized instruments such as the droplet generator, chip loader, and fluorescence microscope.

The study by Crone et al. [66] explored the potential role of non-commercial biofoundries in increasing testing capacity, especially during times of crisis such as the ongoing COVID-19 pandemic [66]. Within a four-week timeframe, they were able to reconfigure existing liquid-handling infrastructure in a biofoundry to establish an automated high-throughput SARS-CoV-2 diagnostic workflow. Compared to manual protocols, automated workflows are preferred as automation not only reduces the potential for human error significantly but also increases diagnostic precision and enables meaningful high-throughput results to be obtained. The modular workflow presented by Crone et al. [66] includes RNA extraction and an amplification setup for subsequent detection by either rRT-PCR, colorimetric RT-LAMP, or CRISPR-Cas13a with a sample-to-result time ranging from 135 min to 150 min. In particular, the RNA extraction and rRT-PCR workflow was validated with patient samples and the resulting platform, with a testing capacity of 2,000 samples per day, is already operational in two hospitals, but the workflow could still be diverted to alternative extraction and detection methodologies when shortages in certain reagents and equipment are anticipated [66].

## 6. Cas13d-Based Assay

The sensitive enzymatic nucleic-acid sequence reporter (SENSR) differed from the abovementioned CRISPR-Cas13-based assays for SARS-CoV-2 detection as the platform uses RfxCas13d (CasRx) from *Ruminococcus flavefaciens*. Similar to LwaCas13a, Cas13d is an RNA-guided RNA targeting Cas protein that does not require PFS and exhibits collateral cleavage activity upon target RNA binding, but Cas13d is ~20% smaller than Cas13a-Cas13c effectors [71]. SENSR is a two-step assay that consists of RT-RPA to amplify the target N or E genes of SARS-CoV-2 followed by T7 transcription and CasRx assay. In addition to designing N and E targeting gRNA, FQ reporters for each target gene were specially designed to contain stretches of poly-U to ensure that the probes were cleavable by CasRx. Collateral cleavage activity was detected either by fluorescence measurement with a real-time thermocycler or visually with an LFD. The LoD of SENSR was found to be 100 copies/µL following 90 min of fluorescent readout for both target genes, whereas the LoD varied from 100 copies/µL (E gene) to 1000 copies/µL (N gene) when visualized with LFD after 1 h of CRISPR-CasRx reaction. A PPA of 57% and NPA of 100% were obtained when the performance of the SENSR targeting the N gene was evaluated with 21 positive and 21 negative SARS-CoV-2 clinical samples. This proof-of-concept work by Brogan et al. [71] demonstrated the potential of utilizing Cas13d in CRISPR-Dx and highlights the possibility of combining Cas13d with other Cas proteins that lack poly-U preference for multiplex detection [71]. However, the low diagnostic sensitivity of SENSR indicated that further optimization is required.

## 7. Cas9-Based CRISPR-Dx

**Figure 3:**
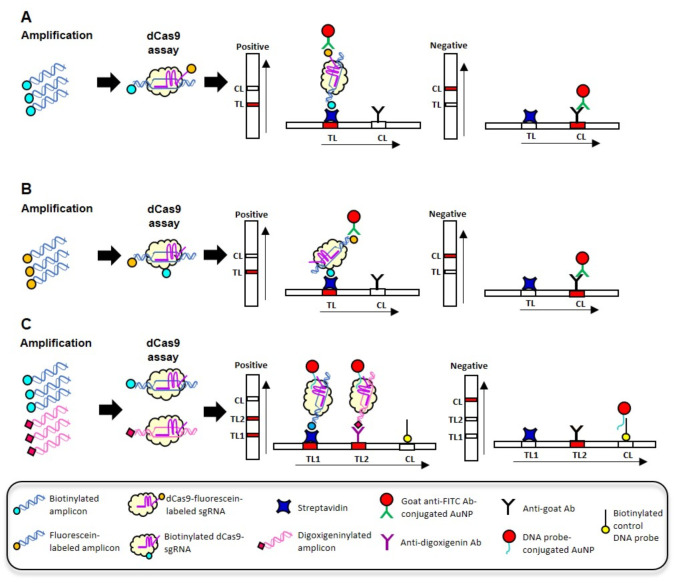
Labeling strategies employed in dCas9-based CRISPR-Dx using LFD for detection. (**A**) The sgRNA is labeled with fluorescein. (**B**) The dCas9 is labeled with biotin. In both (**A**,**B**), the recognition of labeled target amplicons by labeled dCas9-sgRNA results in the formation of a complex containing both biotin and fluorescein labels, allowing the complex to be captured and visualized on an LFD. (**C**) The biotinylated and digoxigeninylated amplicons are specifically captured at different test lines on an LFD. DNA conjugated AuNPs are used as universal label and bind to sgRNA of dCas9-sgRNA. Ab: antibody; AuNP: gold nanoparticles; CL: control line; TL: test line.

The feasibility of utilizing dCas9 for SARS-CoV-2 detection was explored by both Azhar et al. [74] and Osborn et al. [75]. Both assays relied on the visual detection of a labeled dCas9-sgRNA-target DNA complex with a LDF but employed different Cas9 orthologs and labeling strategies. In the FnCas9 Editor-Linked Uniform Detection Assay (FELUDA) developed by Azhar et al. [74], *Francisella novicida* dCas9, and FAM-labeled sgRNA were used to bind with the biotinylated RT-PCR amplicons (nsp8 and N genes) as shown in Figure 3A. FELUDA was shown to be capable of detecting 2 ng of SARS-CoV-2 RNA extract and the total assay time from RT-PCR to result visualization with LFD was found to be ~45 min. In the study by Osborn et al. [75], synthetic SARS-CoV-2 DNA was initially used to demonstrate the specific recognition of the target sequence by dCas9 [75]. Instead of labeled sgRNA, Osborn et al. [75] used biotinylated *Streptococcus pyogenes* dCas9 and unlabeled sgRNA to bind to FAM-labeled, RPA target amplicon (Orf8a gene) (Figure 3B). The 20-min RPA amplification and dCas9 assay were performed sequentially, as combining the steps in a one-pot assay led to non-specific positive results. Alternatively, a competing PAM-rich “soak” DNA was also introduced into the assay to prevent indiscriminate dCas9:DNA interactions that would lead to non-specific DNA labeling and false positive results with the LFD. The authors noted that the test line became more defined with increasing dCas9 assay time and soak DNA concentration. Additional investigation also revealed that single nucleotide resolution of the target DNA could be achieved by using the appropriate soak DNA sequence [75].

Contrary to the findings of Osborn et al. [75], a multiplex one-pot RT-RPA-CRISPR-dCas9 assay was successfully developed by Xiong et al. [76]. During RT-RPA, the E and Orf1ab target genes were amplified simultaneously using biotinylated and digoxigeninylated primers, respectively (Figure 3C). Biotinylated and digoxigeninylated dCas9-sgRNA-target DNA complexes were then generated following incubation with dCas9 and sgRNAs. To differentiate between the complexes, an LFD with two test lines was used wherein the biotinylated complex is captured by the streptavidin-coated test line and the digoxigeninylated complex is captured by the anti-digoxigenin-coated test line. Direct visualization of the captured complexes was afforded by DNA probe-conjugated AuNPs that were housed in the conjugate pad of the LFD. The DNA probe conjugated to AuNPs contained three domains: a binding domain that hybridizes to the scaffold sequence in the loop structure of sgRNA, the middle domain that hybridizes to the probes coated on the control line that serves to capture excess AuNPs, and a poly-A+T domain that is used to functionalize the AuNPs. When combined with a rapid RNA extraction step, the sample-to-result time was estimated to be ~1 h, yielding a LoD of 100 copies/reaction. Evaluation of the RT-RPA-CRISPR-dCas9 assay with 64 clinical nasopharyngeal specimens revealed a PPA of 97% and an NPA of 100% [76].

A colorimetric, microplate-based CRISPR-dCas9 assay that is RNA extraction- and amplification-free was developed for the simultaneous detection of SARS-CoV-2 and influenza A (pH1N1) viruses by exploiting the programmable binding of the dCas9/gDNA complex with PAMmer [77]. To differentiate between the RNA targets of SARS-CoV-2 (N1, N2, and N3) and influenza A (pH1N1 H1) viruses, four types of dCas9-gRNA complexes were individually coated on the microplate well surfaces. Viral lysate, prepared by incubating the specimen in a lysis buffer at 50 °C and 64 °C for 5 min each, was then added with target-specific, biotinylated PAMmer before the mixture was loaded into the dCas9-gRNA-coated wells and incubated at 37 °C for 1 h. After washing and a further incubation at 25 °C for 30 min with streptavidin–horseradish peroxidase (HRP), the presence of the biotinylated PAMmer-target RNA-dCas9-gRNA complex was detected following a washing step and 3,3′,5,5′-tetramethylbenzidine (TMB) substrate addition. The HRP-catalyzed conversion of the colorless TMB into yellowish oxidized TMB can be observed with the naked eye, but an optical density readout can be generated with a microplate reader. The LoD established based on the SARS-CoV-2 N1 target was estimated to be 10 PFU/mL and the assay was shown to be able to detect five SARS-CoV-2 positive samples [77]. In addition to the slight cross-reactivity between SARS-CoV-2 and pH1N1 as noted by the authors, the assay protocol is also more tedious as compared to conventional CRISPR-Dx due to the repetitive incubation and washing steps.

Besides dCas9, Osborn et al. [75] described another strategy to attain multiplex detection using catalytically active Cas9 [75]. To simultaneously detect and differentiate between SARS-CoV-2, influenza A, influenza B, and respiratory syncytial virus (RSV) amplicons, a denaturation/renaturation step is employed to allow virus-specific, distinctive FQ reporters (SARS-CoV-2, FAM; influenza A, TxRed; influenza B, Yakima Yellow; RSV, TAMRA) to hybridize with their complementary target amplicons. Subsequent detection of the heteroduplexes of an amplicon and FQ reporter by the virus-specific Cas9-sgRNA complex would lead to the cleavage of the FQ reporter. The resulting raise in the corresponding fluorescent signal is then detected using a real-time thermocycler. A LoD corresponding to a Ct value of ~35 was obtained for the fluorescent-based Cas9 method, whereas the LoD for the LFD-based method was an order of magnitude below that of the rRT-PCR and fluorescent-based Cas9 method [75].

## 8. Cas3-Based CRISPR-Dx

Yoshimi et al. [31] demonstrated that the collateral cleavage activity of Cas3 could be applied for SARS-CoV-2 detection by developing a platform called Cas3-operated nucleic acid detection (CONAN) [31]. Based on the class I, type 1-E system of *E. coli*, CONAN relies on the recruitment of Cas3 endonuclease by a five-Cas protein complex called Cascade (Cas5, Cas6, Cas7, Cas8, and Cas11) to cleave foreign DNA upon target binding. Following RNA extraction and RT-LAMP at 62 °C for 30 min, the CONAN assay was performed by adding the RT-LAMP amplicon to a CRISPR-Cas3 reaction mixture containing a Cascade-crRNA complex, Cas3, and an ssDNA FQ reporter. The collateral cleavage activity was detected with an LFD after 10 min of incubation at 37 °C. In addition to achieving a LoD of 100 copies, evaluation of the RT-LAMP-CONAN-LFD assay with 10 positive and 15 negative rRT-PCR clinical samples yielded a PPA of 90% and an NPA of 95% [31]. Although CONAN uses an instrument-free approach to visualize the result and a premix of the multiple Cas proteins can be prepared and stored at 4 °C, the instrument and technical requirements in other areas of the workflow would limit the applicability of CONAN-based assays for POC testing. While the collateral cleavage activity of Cas3 has only been discovered recently, we foresee that more Cas3-based CRISPR-Dx will be developed in the near future.

## 9. CRISPR-Cas as an Antiviral Agent

The concept of using CRISPR-Cas to degrade the SARS-CoV-2 genome and to limit its ability to reproduce was first proposed by Nguyen et al. [82]. They chose to focus on Cas13d as its PFS-independent nature confers greater flexibility in gRNA design for target RNA recognition. It would also allow gRNAs to be rapidly developed to target different virus variants, especially those that have evolved to become resistant to existing antiviral drugs. In total, 10,333 gRNAs were designed to target 10 coding regions of the SARS-CoV-2 genome, namely, Orf1ab, S, Orf3a, E, M, Orf6, Orf7a, Orf8, N, and Orf10. As Cas13d is the smallest among the four subtypes of Cas13, the effector can be packaged into one adeno-associated virus (AVV) vector with up to three gRNAs targeting different regions of the SARS-CoV-2 genome to increase the efficiency of virus clearance and for resistance prevention. Although the study did not provide experimental evidence, the authors postulated that tissue-specific promoters could be used to drive the expression of Cas13d in SARS-CoV-2 infected organs, such as the lungs, to functionally disrupt the virus following targeted delivery of the CRISPR system using AAV serotypes that show specific organ tropism profiles [82].

In a separate study, Abbott et al. [83] demonstrated the potential use of a CRISPR-Cas13d-based approach called prophylactic antiviral CRISPR in human cells (PAC-MAN) as a form of genetic intervention against COVID-19 [83]. Similar to Nguyen et al. [80], Cas13d from *R. flavefaciens* was chosen over other Cas13 proteins as Cas13d is not only small (967 amino acids) but it also exhibits high specificity and strong catalytic activity in human cells. Highly conserved regions in the RdRp and N genes of SARS-CoV-2 were firstly identified, allowing 40 crRNAs, with 20 cRNAs targeting each gene, to be designed and synthesized. To test the effectiveness of PAC-MAN, human lung epithelial A549 cells expressing Cas13d through lentiviral infection were first transfected with a SARS-CoV-2 reporter expressing either the RdRp or N gene regions fused to GFP followed by a second transfection with different pools of crRNAs. It was found that the RdRp and N targeting crRNAs pools were able to repress the expression of their corresponding reporter by 81% and 90%, respectively, as compared to a pool of non-targeting control crRNAs. Further bioinformatics analysis revealed that a group of 2, 6, and 22 crRNAs are able to target 50%, 91%, and 100% of the sequenced coronaviruses analyzed (*n* = 3051), respectively. The overall findings of the study indicated that crRNA pools could be used to target different regions of SARS-CoV-2, different SARS-CoV-2 variants, or multiple species of CoVs in order to provide pan-CoV protection [83]. Therefore, the use of a minimal and robust set of crRNAs in PAC-MAN does not only confer a broad level of protection but also guards against the loss of CRISPR-Cas targeting activity due to viral evolution and escape.

Likewise, Wang et al. [84] described a bioinformatic pipeline for the rapid design of a CRISPR-Cas13-based antiviral strategy against SARS-CoV-2, but proposed an alternative time-saving approach wherein a unique region is first identified followed by crRNA design instead of screening and testing a large number of potential crRNA sequences [84]. In contrast to the work by Abbott et al. [81], Wang et al. [84] utilized LwaCas13a from *L. wadeii*, targeted the S protein region, and tested the antiviral tool in human hepatocarcinoma (HepG2) and human alveolar epithelial type II (AT2) cells. Despite the high nucleotide sequence identity of the S protein between SARS-CoV-2 and SARS-CoV (76–78%), two unique regions in the receptor binding domain of SARS-CoV-2 were identified and one of the regions was selected due to the potentially greater structural variation between the two species. HepG2 and AT2 expressing Cas13a through lentiviral infection were first transfected with a plasmid expressing GFP-fused S protein and later with crRNAs. Out of the 12 crRNA that were designed, the rRT-PCR results showed that only crRNA-6 exhibited a significant cleavage-guiding effect with high knockdown efficiencies in both HepG2 (>99.9%) and AT2 (>93%) cells. Proteomic analysis of Cas13a-crRNA-6 in AT2 cells expressing the S protein revealed a significant decrease in total proteins, especially the S and ACE proteins, as compared to the control group. They also explored the potential use of catalytically dead dCas13a and found that dCas13a-crRNA6 did not affect cell viability but was able to reduce the S protein expression without altering its RNA expression level. Based on these findings, Wang et al. [84] proposed two potential therapeutic strategies for COVID-19: (1) eradication of viral genome with CRIPSR-Cas13a during early stage infection with simultaneous injury and death to the infected cells due to the collateral cleavage activity of Cas13a and (2) reduction of viral protein synthesis with CRISPR-dCas13a during late stage infection without cytotoxicity effect to the infected cells [84].

## 10. Summary and Perspectives

Rapid testing is important, not only to curb the current COVID-19 pandemic, but also in future outbreak settings where it will be instrumental in early detection and implementation of infection control measures. Diagnostic technologies that are highly sensitive and specific as well as easily customized are ideal platforms on which new tests can be rapidly developed, validated, and deployed for clinical use during a public health crisis. It is not surprising that rRT-PCR is deemed as the “gold standard” for COVID-19 testing since the method is well established and highly versatile. Primers and probes can be designed to target virtually any nucleic acid sequence, but the rRT-PCR instrument and skill personnel requirements hamper its implementation and use in POC settings [17,85,86,87,88]. The difficulty in implementing a new rRT-PCR test in hospital laboratories, especially under the constraints of a pandemic, has led to invalid and inconclusive results being obtained [40], and this can hinder the timely initiation of appropriate patient management. Through next-generation sequencing, a new pathogen and its variants can be rapidly identified and, more importantly, it fuels the development of alternative nucleic acid-based diagnostic tools and therapeutic options afforded by emerging technologies such as the highly programmable CRISPR-Cas system.

The majority of CRISPR-Dx for COVID-19 exploit isothermal amplification techniques such as RT-LAMP, RT-RPA, and RT-RAA to efficiently amplify the target sequence, to shorten the assay time, and to eliminate the use of specialized instruments such as the thermocycler. At the time of writing, various strategies have been described to streamline the workflow and to improve the performance of CRISPR-Dx for COVID-19, including the following: (1) direct detection of SARS-CoV-2 without RNA extraction and amplification; (2) a simple specimen processing step such as a heat lysis method to circumvent the RNA extraction step [42,59,61,62,63]; (3) a one-pot system that allows the target amplification and Cas assay to be conducted in a closed-tube format [52,53,54,55,56,57]; (4) enhancement in assay sensitivity through the use of engineered crRNA or Cas protein, divalent cation, and light-up aptamer [64,65,81]; (5) strategies to minimize mutational escape and to achieve multiplex detection [35,50,52,54]; (6) chip-based testing that reduces sample and reagent volumes [42,58,59]; (7) the fabrication of portable and low-cost instrument using 3D printing technology with potential POC applications; (8) result interpretation that leverages smartphone imaging and cloud-based analysis [36,53]; and (9) a fully automated platform for high-throughput testing [66,67]. Nonetheless, most of these CRISPR-Dx platforms were presented as proof-of-concept, and validation efforts may have been hindered by the lack of access to SARS-CoV-2-positive specimens during the early phase of the outbreak. Hence, further emphasis on analytical and clinical validation will be required if these platforms are to gain widespread acceptance as diagnostic tools.

At present, the number of CRISPR-Cas12-based assays developed to detect SARS-CoV-2 exceeds that of Cas13-, Cas9-, and Cas3-based assays. The CRISPR-Dx platforms developed with Cas12, Cas13, and Cas3 take advantage of the non-specific collateral cleavage activity that is activated upon the recognition of its target sequence, but this inherent feature also presents a challenge when multiple targets are to be simultaneously detected and differentiated in a single reaction. Some researchers opted to set up separate reactions in different tubes or microwells in order to detect multiple target genes, but such an approach will inadvertently increase the volume of sample needed, the number of liquid handling steps, the assay cost, and the turnaround time [14,17,89,90]. One of the possible strategies to overcome this predicament is to use a combination of different Cas proteins, such as PsmCas13b, LwaCas13a, CcaCas13b, and AaCa12a, in a single reaction [91]. As each Cas protein has its own sequence preference and the corresponding FQ reporters can be labeled with a distinct fluorophore, the fluorescence emission detected at the end point will allow the target sequences to be distinguished. However, the multiplex capability will be limited by the types of Cas proteins that can be combined in a single reaction. Likewise, the Cas9-based multiplexing approach described by Osborn et al. [75] is limited by the fluorescence channels of the real-time thermocycler used while the dCas9-based multiplexing approach described by Xiong et al. [76] is limited by the hapten–antibody combinations. Future exploration into sequence-specific hybridization-based LFD [92] or even digital multiplexing, as exemplified by the πCode MicroDisc [93] and barcoded magnetic beads [94] technologies, may be possible avenues to expand the multiplexing potential of CRISPR-Dx.

CRISPR-Dx, with its short assay time, also holds the potential to decentralize testing when combined with low-cost, highly portable instrumentation while retaining high sensitivity and specificity. The modular nature of CRISPR-Dx also makes it amenable to large scale, high-throughput testing as well as low-throughput and even home-based testing. Future research in CRISPR-Dx may also be directed towards the development of closed systems with sample-to-result functionality that could be geared towards mass testing or POC testing. Lyophilized CRISPR-Cas reagents that are stable at room temperature could be developed to eliminate the dependency on cold chain storage and transport. The development of CRISPR-Dx with the ability to quantitate viral load has also lagged behind that of qualitative-based CRISPR-Dx. As demonstrated by Fozouni et al. [70], SARS-CoV-2 viral load quantification could be achieved with an amplification-free, CRISPR-Cas13-based assay [70], but this area of CRISPR-Dx research is considerably less explored. As rapid advancement continues to transform the CRISPR-Cas technology, it is inevitable that CRISPR-Dx will rise to become one of the mainstream platforms in the future and may even play a central role in minimizing the devastating impact of future unprecedented pandemics.

Compared to vaccines and conventional therapies that provoke the human immune system to recognize and destroy the viral proteins, the CRISPR-Cas system exerts its antiviral effects by searching for and destroying the mRNAs and RNA genome of SARS-CoV-2 to impede protein expression and viral replication. While the emergence of new variants poses the risk of immune escape and threatens the efficacy of existing vaccines, the CRISPR-Cas-based antiviral therapy can be tweaked by changing or incorporating new crRNAs to compensate for the loss of targeting activity. Other than targeting the SARS-CoV-2 genome with the CRISPR-Cas system to impair its activity or to abolish the virus from the host, host-based interventions have been proposed as a more promising alternative for viral eradication. By targeting the host cell machinery instead of the viral genome, the chance of drug resistance development is lowered due to the genetic stability of host factors, and extension of the therapeutic time frame would also increase the treatment efficacy while side effects would be reduced through the minimal dosing requirements [95]. Recent transcriptional studies involving SARS-CoV-2-, SARS-CoV-, and MERS-CoV-infected cells have identified key host genes that were involved in host-pathogen interactions [96,97,98]. More importantly, the upregulated genes that were found to play a crucial role in disease progression represent potential CRISPR-Cas targets for the development of therapeutics.

Despite the prophylactic and therapeutic potentials of the CRISPR-Cas system, there are several challenges that need to be overcome before the technology becomes suitable for clinical applications [83,84]. Firstly, the CRISPR-Cas antiviral strategies need to be tested with live SARS-CoV-2 virus in live cell model and secondly, a safe and effective CRISPR-Cas in vivo delivery method into the target human epithelial cells has to be established. Several delivery systems, such as AAV, lipid nanoparticles, chemical polymers, amphiphilic peptide, and liposome, have been proposed as viable options. Furthermore, the dosage and timing of the delivery have to be optimized as the CRISPR-Cas system will only work if it is sufficiently expressed in the host cells. Lastly, the specificity, efficacy, and risk of immunogenicity of the CRISPR-Cas system have to be validated in animal models before moving on to clinical trials. With next generation sequencing technology, the off-target effects of the CRISPR-Cas system can be easily identified through whole transcriptomic RNA sequencing.

In conclusion, these works support and provide new insight into the growing potential of CRISPR-Cas in revolutionizing diagnostics, prophylaxis, and therapeutics. In the course of this COVID-19 pandemic, the CRISPR-Cas system has opened up new opportunities in both diagnostics and therapeutics as evidenced by the surge in the development of various CRISPR-Dx tools and therapeutic strategies. The CRISPR-Cas-based strategies that are presented as proof-of-concept will neither result in immediate clinical utility nor suppress the uprising tide of COVID-19 infections. However, the groundwork that has been laid and the continual progress achieved in the expansion of CRISPR-Cas-based applications will be invaluable in the fight against future viral threats or the next pandemic.

## Figures and Tables

**Table 1:** Characteristics of representative Cas proteins used in CRISPR-Dx for COVID-19.

	CRISPR-Cas12a	CRISPR-Cas13a	CRISPR-Cas3	CRISPR-Cas9
**Class**	2	2	1	2
**Type**	V	VI	I	II
**Effector Cas protein complex**	Single unit	Single unit	Multi-subunit	Single unit
**Size (amino acid)**	~1200 (LbCas12a)	~1200 (LwaCas13a)	~900 (EcoCas3)	~1400 (SpCas9)
**Nuclease domain**	RuvC	2 HEPN domains	HD	RuvC, HNH
**PAM/PFS**	5′ T-rich PAM	3′ non-G PFS	Variable PAM (recognition by Cascade)	3′ G-rich PAM
**Pre-crRNA processing**	Yes	Yes	Yes	No
**tracrRNA**	No	No	No	Yes
**On target substrate (activator)**	ssDNA, dsDNA	ssRNA	dsDNA	dsDNA (ssDNA and ssRNA with PAMmer)
**Collateral cleavage activity**	Yes	Yes	Yes	No
**Off target substrate**	ssDNA	ssRNA	ssDNA	NA

**Table 2:** Characteristics of various CRISPR-Dx for COVID-19.

Cas Protein	Assay Name	RNA Extraction	Assay Component	Time Required ^a^	Target Gene(s)	Result Interpretation	LoD	PPA (n) ^b^	NPA (n) ^b^	Ref.
**Cas12-based CRISPR-Dx**										
LbCas12a	SENA	Yes	rRT-PCR (~64 min); Cas12 (48 °C, 10 min)	~74 min	N, Orf1ab	FR (real-time thermocycler)	1.6 copies/reaction	92% (24 ^d^)	98% (240 ^e^)	[41]
LbCas12a	COVID-19 CRISPR-FDS	Yes	RT-PCR (~38 min)/RT-RPA (42 °C, 20 min); Cas12 (37 °C, 20 min)	40–60 min	N, Orf1ab	FR (plate reader)	2 copies/reaction	100% (15)	71.4% (14)	[40]
LbCas12a	SARS-CoV-2 RNA DETECTR	Yes	RT-LAMP (62 °C, 30 min); Cas12 (37 °C, 15 min)	45 min	N	FR (real-time thermocycler)	20 copies/µL	95% (40)	100% (62)	[47]
LbCas12a	DETECTR	Yes	RT-LAMP (62 °C, 20–30 min); Cas12 (37 °C, 10 min)	30–40 min	E, N2	FR (plate reader), LFD	10 copies/µL	95% (36)	100% (42)	[14]
LbCas12a	DETECTR	Yes	RT-LAMP (62 °C, 20 min); Cas12 (37 °C, 10 min)	30 min	N	FR (plate reader), LFD	50 copies (plasmid)	93% (155)	96% (223)	[35]
LbCas12a	-	Yes	RT-RPA (42 °C, 30 min); Cas12 (37 °C, up to 90 min)	60–120 min	Orf1ab	FR (plate reader), LFD	10 copies/µL	-	-	[48] ^†^
LbCas12a	CRISPR-Cas12a-NER	Yes	RT-RAA (39 °C, 30 min); Cas12 (37 °C, 15 min)	45 min	E	Visual (under blue light)	10 copies	100% (16)	100% (15)	[49]
LbCas12a	-	Yes	RT-RPA (39 °C, 30 min; 75 °C, 5 min); Cas12 (39 °C, 15 min)	50 min	S	Visual (under blue light)	10 copies/reaction	96% (53)	100% (111)	[50]
LbCas12a	-	Yes	RT-RPA (39 °C, 30 min; 75 °C, 5 min); Cas12 (39 °C, 30 min)	65 min	N	Cloud-based analysis (smartphone-based FR device)	6.25 copies/µL	87% (52)	92% (63)	[36]
LbCas12a	Two-pot iSCAN	Yes	RT-LAMP (62 °C, 30 min); Cas12 (37 °C, 10 min)	40 min	E, N	Visual (under UV light), LFD	10 copies/reaction	86% (N, 21); 38% (E, 21)	100% (N, 3); 100% (E, 3)	[51]
LbCas12a	AIOD-CRISPR	Yes	RT-RPA and Cas12 (37 °C, 40 min)	40 min	N	Visual (under blue/UV light)	~ 5 copies	100% (8)	100% (20)	[52]
LbCas12a	-	Yes	RT-LAMP (65 °C, 40 min); Cas12 (37 °C, 5 min)	45 min	Orf	Visual (smart phone-based FR device)	20 copies/reaction	100% (7)	100% (3)	[53]
LbCas12a	opvCRISPR	Yes	RT-LAMP (65 °C, 40 min); Cas12 (37 °C, 5 min)	45 min	S	Visual (under blue light)	5 copies	100% (26)	100% (24)	[54]
LbCas12a	-	Yes	RT-LAMP (62 °C, 30 min); Cas12a (r.t., 10 min)	40 min	E, N	Visual (under UV light)	30 copies/µL (N); 45 copies/µL (E)	94% (50)	100% (50)	[55]
LbCas12a	OR-DETECTR	Yes	RT-RPA (42 °C, 30 min); Cas12 (42 °C, 20 min)	50 min	N, RdRp	FR (plate reader), LFD	Synthetic RNA: 10 copies/µL (RdRp), 2.5 copies/µL (N); pseudo-virus: 20 copies/µL (RdRp), 1 copy/µL (N)	100% (6)	100% (8)	[56]
AapCas12b	CASdetec	Optional (lysis: 50 °C, 5 min; 64 °C, 5 min)	RT-RAA (42 °C, 30 min); Cas12 (42 °C, 30 min)	60 min	RdRp	FR (real-time thermocycler), visual (under blue LED)	FR: 1 × 10^4^ copies/mL; visual: 5 × 10^4^ copies/mL (pseudo-virus)	-	-	[57]
AapCas12b	STOPCovid. v2	Yes (lysis and magnetic bead-based purification: r.t., 10 min)	RT-LAMP and Cas12 (60 °C, 45 min for FR/80 min for LFD)	45–80 min	N	FR (real-time thermocycler), LFD	FR: 33 copies/mL; LFD: 83 copies/ml	93% (202)	99% (200)	[37]
AapCas12b	One-pot iSCAN	Yes	RT-LAMP (62 °C, 30 min); Cas12 (62 °C, 15 min)	60 min	N	Visual (under UV light), LFD	10 copies/reaction	86% (N, 21)	100% (N, 3)	[51]
LbCas12a	ITP-CRISPR	Yes (Off-CHIP, 95 °C, 2 min; on-chip, 3 min)	RT-LAMP (off-chip, 62 °C, 30 min); Cas12a (on-chip, 5 min)	35 min	E, N	Fluorescent microscopy	10 copies/µL	94% (32)	100% (32)	[58]
LbCas12a	deCOViD	Optional (heat inactivation: 65 °C, 30 min)	RT-RPA and Cas12a (42 °C, 30–60 min)	30–60 min	N	Fluorescent microscopy	Synthetic RNA: 1 GE/µL; heat-inactivated virus: 20 GE/µL	100% (2)	100% (2)	[59]
LbCas12a	CRISPR-FDS	No (lysis: 37 °C, 5 min)	RT-RPA & Cas12 (r.t., 10 min)	15 min	Orf1ab	Smartphone-based fluorescent microscopy	0.38 copies/µL	-	-	[42]
LbCas12a	-	No (lysis: 65 °C, 10 min)	RT-LAMP (65 °C, 30 min); Cas12a (37 °C, 10–20 min)	40–50 min	Orf	Visual (under UV light)	20 copies/reaction (pseudo-virus)	100% (4)	100% (4)	[60]
LbCas12a	-	No (lysis: 80 °C, 5 min)	RT-RPA (42 °C, 15–20 min); Cas12 (37 °C, 15-20 min)	30–40 min	N, Orf1ab	Visual (under UV light), LFD	Visual: 1 copy/reaction LFD: 1 copy/µL	100% (11)	100% (11)	[61]
LbCas12a	-	No (lysis: 42 °C, 20 min; 64 °C, 5 min)	RT-LAMP (65 °C, 30 min); Cas12 (37 °C, 10 min)	40 min	N	FR (real-time thermocycler), Visual (under blue light)	16 copies/µL	100% (6)	100% (6)	[62]
enAsCas12a	VaNGuard (quasi-one-pot)	Optional (proteinase K and heat treatment: 95 °C, 5 min)	RT-LAMP (60 °C/63 °C, 22 min); Cas12 (60 °C, 5 min)	27 min	S	LFD	RNA extract: 2 copies/µL; proteinase K and heat treatment: 40 copies/µL	RNA extract: 84% (51); proteinase K and heat treatment: 76% (21)	RNA extract: 100% (36); proteinase K and heat treatment: 100% (21)	[63]
LbCas12a	MeCas12a	Yes	RT-RAA (39 °C, 30 min); desalting (~3 min); Cas12 (37 °C, 15 min)	45 min	E	Visual (under blue light)	5 copies	100% (13)	100% (11)	[64]
LbCas12a	CRISPR-ENHANCE	Yes	RT-LAMP (63 °C, 20–30 min); Cas12 (37°, 20 min)	40–50 min	N	LFD	3–300 copies	-	-	[65]
**Cas13-based CRISPR-Dx**										
LwaCas13a	SHERLOCK	Yes	RT-RPA (42 °C, 25 min); T7 transcription and Cas13 (37 °C, 30–60 min for FR/30 min for LFD)	55–85 min	S	FR (plate reader/real-time thermocycler), LFD	42 copies/reaction	FR: 96% LFD: 8% (81)	FR: 100% LFD: 88% (73)	[38]
Cas13a	CRISPR-COVID	Yes	RT-RPA (42 °C, 30 min); T7 transcription and Cas13 (42 °C, 10 min)	40 min	Orf1ab	FR	7.5 copies/reaction (plasmid)	100% ** (52)	100% ** (62)	[39]
LwaCas13a	-	Yes	RT-PCR (~40 min)/RT-RPA (42 °C, 30 min); T7 transcription and Cas13 (37 °C, 120 min)	150–160 min	N (RT-PCR); Orf1ab (RT-RPA)	FR (plate reader)	N: ~2.5 copies/reaction Orf1ab: ~200 copies/reaction (Virus-like particle)	-	-	[66]
LwaCas13a	CARMEN	Yes	Complex workflow	(SAMPLE-to-result: ~6.5 h)	-	Fluorescent microscopy	-	-	-	[67]
LwaCas13a	CREST	Optional (PEARL: 25 min)	RT (42 °C, 30 min); PCR (22 min); Cas13 (37 °C, 5–30 min)	57–82 min	N1, N2, N3	Visual (under blue light)	10 copies/µL	97% (65)	98% (153)	[68]
LwaCas13a	-	Yes	Ligation (37 °C, 30 min); Transcription amplification (37 °C, duration not specified); Cas13 (37 °C, 20 min)	-	E, N	FR	82 copies (pseudo-virus)	100% (5)	100% (1)	[69]
LbuCas13a	-	Yes	Cas13 (37 °C, 30 min)	30 min	E, N	Smartphone-based fluorescent microscopy	~100 copies/µL	100% (5)	-	[70]
RfxCas13d (CasRx)	SENSR	Yes	RT-RPA (42 °C, 45 min); T7 transcription and Cas13 (37 °C, 90 min for FR/60 min for LFD)	105–135 min	E, N	FR (real-time thermocycler), LFD	~100 copies/µL	57% (21)	100% (21)	[71] ^†^
LwaCas13a	SHINE	No (HUDSON: 40 °C, 5 min; 70 °C (95 °C for saliva), 5 min)	RT-RPA, T7 transcription and Cas13 (37 °C, 40 min)	40 min	Orf1a	FR (under blue light with a smartphone application), LFD	FR: 10 copies/µL LFD: 100 copies/µL	90% (30)	100% (20)	[43]
LwaCas13a	ERASE	Yes	RT-RAA (42 °C, 30 min); T7 transcription and Cas13 (37 °C, 30 min)	60 min	N	LFD	1 copy/µL	91% (286)	99% (379)	[72]
LwaCas13a	Sherlock CRISPR SARS-CoV-2 kit	Yes	RT-LAMP (61 °C, 40 min); T7 transcription and Cas13 (37 °C, 10 min)	50 min	N, Orf1ab	FR (plate reader)	6.75 copies/µL (Orf1ab); 1.35 copies/µL (N)	100% (30)	100% (30)	[73]
**Cas9-based CRISPR-Dx**										
dFnCas9	FELUDA	Yes	RT-PCR (42 min); dCas9 (37 °C, 10 min)	52 min	N, S	LFD	10 copies	100% (21)	97% (60)	[74] ^†^
dSpCas9	-	-	RPA (r.t., 20 min); dCas9 (37 °C, 60 min)	80 min	Orf8a	LFD	50 copies (synthetic DNA)	-	-	[75]
SpCas9	-	-	Hybridization (95 °C, 5 min; cooling to r.t. at -0.1 °C/s); Cas9 (37 °C, 60 min)	~77 min	Orf8a	FR (real-time thermocycler)	5 copies (synthetic DNA)	-	-	[75]
dSpCas9	-	Yes	Multiplex RT-RPA (37 °C, 30 min); dCas9 (37 °C, 5 min)	35 min	E, Orf1ab	LFD	100 copies/reaction	97% (35)	100% (29)	[76]
dSpCas9	-	No (Lysis: 50 °C, 5 min; 64 °C, 5 min)	Complex workflow	(Sample-to-result: 90 min)	N1, N2, N3	Colorimetry (plate reader)	10 PFU/ml	100% (5)	100% (3)	[77]
**Cas3-based CRISPR-Dx**										
EcoCas3	CONAN	Yes	RT-LAMP (62 °C, 30 min); CONAN (37 °C, 10 min)	40 min	N1, N2	LFD	100 copies	-	-	[31] ^†^

^a^ Time required for nucleic acid amplification and Cas assay; ^b^ performance relative to rRT-PCR; ^c^ performance relative to metagenomic NGS; ^d^ two rRT-PCR results were confirmed to be false positive with NGS; ^e^ three rRT-PCR results were confirmed to be false negative with NGS; ^†^ preprint articles. PPA: positive percent agreement, NPA: negative percent agreement, FR: fluorometry, LFD: lateral flow device, r.t.: room temperature, PEARL: precipitation enhanced analyte retrieval, HUDSON: heating unextracted diagnostic samples to obliterate nucleases.
